# An Essay on the Nature and Treatment of Apoplexy

**Published:** 1843-07

**Authors:** 


ART. VII.—*An Essay on the Nature and Treatment of Apoplexy.* By M. GAY.—*Paris,* 1808. Translated by EDWARD COPEMAN, Surgeon. *With an Appendix.—London,* 1843. 8vo, pp. 94.

MR. COPEMAN, after having been struck with the erroneousness of the too common practice of bleeding in all cases of apoplexy without regard to the concomitant symptoms, and having found the advantage of substituting emetics in cases where the attack had followed a full meal, and purgatives where there was no other indication—met with a treatise on Apoplexy published in Paris in 1808, and has translated it. M. Gay first combats with much warmth and intelligence the views of Portal, the authority of that day, who recommends bleeding in apoplexy; and then proposes to prove that *no* such disease as sanguineous apoplexy exists, that bleeding is injurious in *all* cases of apoplexy, and that emetics are indicated in *every case* because the primary cause is *always* in the primæ viæ. This is proved by reasoning, the force of which we confess our inability to understand; and having seen clots of blood in the brain, having witnessed the occasional good effects of bleeding, and having seen a large clot in the brain following the act of stooping, we should suspect a fallacy, even in an argument which seemed logically to prove that no such things can happen. Emetics have been recommended by the highest authorities, and when the attack follows a full meal, the administration of an emetic is undoubtedly judicious; and there are few cases we suspect where free purging is not indicated. That bleeding is too indiscriminately used cannot be doubted, and it is not improbable that it is often carried so far as to deprive nature of her power of remedying the injury which has already taken place. There are no cases in which bleeding is more frequently resorted to on account of the name of the disease, and not from the actual symptoms of the particular case, always an empirical proceeding, and here much encouraged by the wishes of lookers-on. The difficulty is to lay down rules. Mr. Copeman in an appendix has reprinted two papers on this subject, originally published in the Medical Gazette, which well merit the attention of practitioners. And we cannot but regret that one who could write so judiciously himself should have taken the pains to translate the work of a writer so much his inferior in observation, judgment, and good sense. With such qualities of mind, and with opportunities of seeing disease, we doubt not but that Mr. Copeman may, if he chooses, be of general service by communicating the results of his own observations and reflections on the treatment of many diseases.

